# Effect of Synchronous Versus Sequential Regimens on the Pharmacokinetics and Biodistribution of Regorafenib with Irradiation

**DOI:** 10.3390/pharmaceutics13030386

**Published:** 2021-03-13

**Authors:** Tung-Hu Tsai, Yu-Jen Chen, Li-Ying Wang, Chen-Hsi Hsieh

**Affiliations:** 1 Institute of Traditional Medicine, School of Medicine, National Yang Ming Chiao Tung University, Taipei 112, Taiwan; thtsai@ym.edu.tw (T.-H.T.); chenmdphd@gmail.com (Y.-J.C.); 2 Departments of Radiation Oncology, Mackay Memorial Hospital, Taipei 104, Taiwan; 3 Department of Medical Research, China Medical University Hospital, Taichung 404, Taiwan; 4 Department of Nursing, MacKay Junior College of Medicine, Nursing and Management, Taipei 112, Taiwan; 5 School and Graduate Institute of Physical Therapy, College of Medicine, National Taiwan University, Taipei 100, Taiwan; liying@ntu.edu.tw; 6 Physical Therapy Center, National Taiwan University Hospital, Taipei 100, Taiwan; 7 Faculty of Medicine, School of Medicine, National Yang Ming Chiao Tung University, Taipei 112, Taiwan; 8 Division of Radiation Oncology, Department of Radiology, Far Eastern Memorial Hospital, New Taipei City 220, Taiwan

**Keywords:** biodistribution, pharmacokinetics, radiotherapy, regorafenib, stereotactic body radiation therapy (SBRT)

## Abstract

This study was performed to evaluate the interaction between conventional or high-dose radiotherapy (RT) and the pharmacokinetics (PK) of regorafenib in concurrent or sequential regimens for the treatment of hepatocellular carcinoma. Concurrent and sequential in vitro and in vivo studies of irradiation and regorafenib were designed. The interactions of RT and regorafenib in vitro were examined in the human hepatoma Huh-7, HA22T and Hep G2 cell lines. The RT–PK phenomenon and biodistribution of regorafenib under RT were confirmed in a free-moving rat model. Regorafenib inhibited the viability of Huh-7 cells in a dose-dependent manner. Apoptosis in Huh-7 cells was enhanced by RT followed by regorafenib treatment. In the concurrent regimen, RT decreased the area under the concentration versus time curve (AUC)_regorafenib_ by 74% (*p* = 0.001) in the RT_2 Gy × 3 fraction (f’x)_ group and by 69% (*p* = 0.001) in the RT_9 Gy × 3 f’x_ group. The AUC_regorafenib_ was increased by 182.8% (*p* = 0.011) in the sequential RT_2Gy × 1 f’x_ group and by 213.2% (*p* = 0.016) in the sequential RT_9Gy × 1 f’x_ group. Both concurrent regimens, RT_2Gy × 3 f’x_ and RT_9Gy × 3 f’x_, clearly decreased the biodistribution of regorafenib in the heart, liver, lung, spleen and kidneys, compared to the control (regorafenib _× 3 d_) group. The concurrent regimens, both RT_2Gy × 3 f’x_ and RT_9Gy × 3 f’x_, significantly decreased the biodistribution of regorafenib, compared with the control group. The PK of regorafenib can be modulated both by off-target irradiation and stereotactic body radiation therapy (SBRT).

## 1. Introduction

The incidence of hepatocellular carcinoma (HCC) and the associated mortality are increasing in North America and several European regions and declining in Japan and parts of China [1]. The oral multikinase inhibitor sorafenib (Nexavar, Bayer Pharma AG, Berlin, Germany) is the first-line systemic treatment and has shown clinical benefits in overall survival [2,3]. However, most HCC patients experience disease progression during sorafenib treatment, and the overall survival is approximately eight months [4,5,6]. Recently, regorafenib (Stivarga, BAY 73-4506; Bayer Pharma AG, Berlin, Germany), a diaryl urea derivative, was shown to provide a survival benefit for HCC patients after progression during sorafenib treatment [7]. Therefore, the National Comprehensive Cancer Network^®^ Clinical Practice Guidelines in Oncology (NCCN Guidelines^®^) listed regorafenib as a category 1 systemic therapy for patients with HCC progression on or after sorafenib.

External beam radiotherapy (EBRT) with 3D conformal radiotherapy, intensity-modulated radiotherapy (IMRT) or stereotactic body radiation therapy (SBRT) are options for patients with unresectable or medically inoperable disease, as listed in the NCCN Guidelines^®^. SBRT is an advanced technique of hypofractionated EBRT, referring to the use of focused high dose radiation generally delivered in five or fewer treatment sessions with image-guided technique [8]. Dosing for SBRT is generally 30–50 Gy in three to five fractions. Radiotherapy (RT) combined with sorafenib therapy in patients with unresectable HCC has been reported to show impressive benefits [9,10]. However, more than a 30% incidence of adverse effects has been reported for HCC patients who received RT concurrently or sequentially with sorafenib [11,12,13]. Recently, a patient was reported to have developed transverse myelopathy from regorafenib two years after receiving SBRT for metastatic liver lesions [14]. However, a colon cancer patient who received regorafenib and concurrent SBRT for an oligometastatic lung nodule showed an impressive response [15]. The treatment or toxicities of regorafenib with RT have different expressions in the concurrent or sequential regimen. Additionally, the target dose and off-target dose or conventional dose of RT can modulate the systemic pharmacokinetics (PK) in a rat model [10,16,17]. These lines of evidence suggest interactions between RT and diaryl urea agents, such as sorafenib and regorafenib.

In the current study, the RT–PK behavior of regorafenib at different RT doses and time schedules was evaluated in a free-moving rat model and verified in the human hepatoma Huh-7, HA22T and Hep G2 cell lines. Furthermore, the biodistribution of regorafenib with and without RT was evaluated, to provide suggestions for clinical applications.

## 2. Materials and Methods

### 2.1. Materials and Reagents

#### Reagents

Regorafenib (BAY 73–4506) was purchased from Toronto Research Chemicals, Inc. (Ontario, Canada). The chemical purity was >99% (data provided by Toronto Research Chemicals, Inc.). Dimethyl sulfoxide (DMSO) and 3-(4′,5′-dimethylthiazol-2′-yl)-2,5-diphenyltetrazolium bromide (MTT) were purchased from Merck (Merck Ltd., Taiwan). Dulbecco’s modified Eagle medium (DMEM), fetal bovine serum, 100 IU/mL penicillin, 100 mg/mL streptomycin and 1% nonessential amino acids were purchased from Biological Industries (Cromwell, CT, USA). Milli-Q plus water (Millipore, Bedford, MA, USA) was used for all preparations. For cell culture experiments, regorafenib was dissolved in DMSO at various concentrations and then added to cells in serum-free DMEM and stored at 4 °C. A stock solution of 5 mg/mL MTT in PBS was stored at −20 °C.

### 2.2. In Vivo Study

#### 2.2.1. Animals and Sample Preparation

The Institutional Animal Experimentation Committee of National Yang-Ming University, Taipei, Taiwan, and the Institutional Animal Care and Use Committee (IACUC, approval number 1070523) reviewed and approved the protocol. The Laboratory Animal Center at National Yang-Ming University (Taipei, Taiwan) provided adult male Sprague-Dawley rats (300 ± 20 g body weight). A pathogen-free environment with a 12 h light–dark cycle, with access to water ad libitum and food (laboratory rodent diet 5P14, PMI Feeds, Richmond, IN, USA), was provided for the animals.

#### 2.2.2. Irradiation Technique

A freely moving rat model was designed for the current study [10]. The rats were anaesthetized and immobilized on a board, while undergoing computed tomography for localization of the whole liver or a central area 1.5 cm × 1.5 cm in size for the SBRT technique. For the whole liver field, the cranial margin was set 5 mm from the top of the diaphragm, and the caudal margin was set 5 mm lower than the liver margin. The whole liver was targeted for irradiation. The experimental animals were randomized to groups receiving sham RT, one or three fractions of RT_2 Gy_ and RT_9 Gy_ with current or sequential regorafenib. Data were collected from six rats in each group.

#### 2.2.3. Drug Delivery with RT under Different Time Schedules and Doses

**Figure 1:**
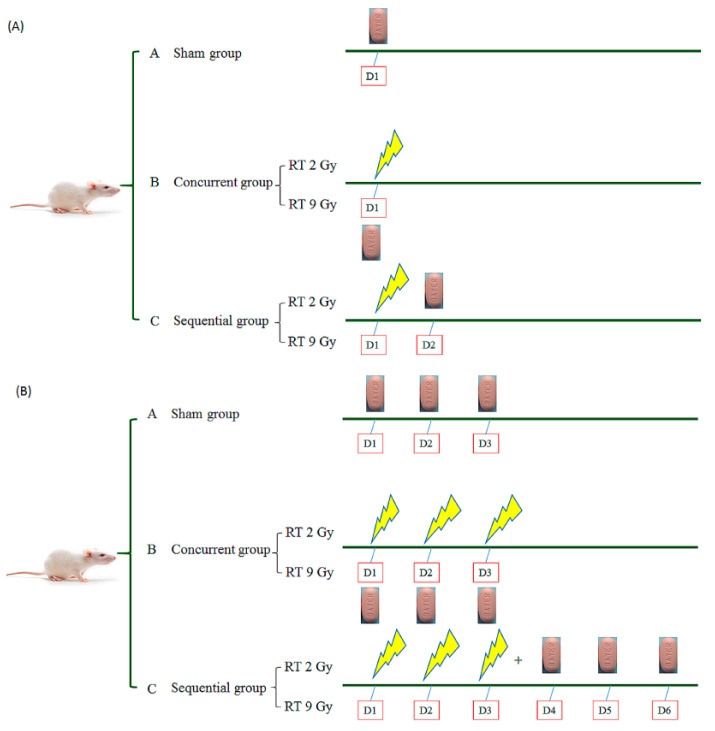
Oral regorafenib 16 (mg/kg/day) delivery with irradiation (i.e., radiotherapy (RT)), under different time schedules and RT doses. (**A**) The one fraction study groups; (**B**) The continue treated groups. The rats were randomly divided into ten groups with six rats in each group.

The administration of oral regorafenib (160 mg) once daily as a systemic treatment has been shown to provide survival benefit in HCC patients progressing on sorafenib treatment [7]. According to the formula used to translate doses from animal to human, human equivalent dose (HED, mg/kg) = animal dose (mg/kg) × animal km/human km [18], we calculated the daily dose of regorafenib for rats to be 16 (mg/kg/day). The rats were randomly divided into ten groups with six rats in each group. The concurrent groups treated with regorafenib 1 h after RT, in the same day, to mimic regorafenib concurrent with RT in the daily practice. The sequential groups treated with regorafenib following RT (not in the same day), to mimic RT followed by regorafenib in the daily practice. The one fraction study group included (A) a sham group, regorafenib with RT_0 Gy_ (regorafenib × 1 d); concurrent groups treated with regorafenib 1 h after (B) RT_2 Gy_ with 1 fraction (RT_2 Gy × 1 f’x_) and (D) RT_9 Gy_ with 1 fraction (RT_9 Gy × 1 f’x_); and sequential groups treated with regorafenib 24 h after (C) RT_2 Gy × 1 f’x_ and (E) RT_9 Gy × 1 f’x_. Continued treatment mimicking clinical practice was applied to (A) a sham group, regorafenib (p.o. (per os), q.d. × 3 d) with RT_0 Gy_ (regorafenib × 3 d); concurrent groups treated with regorafenib (p.o., q.d. × 3 d) 1 h after (B) RT_2 Gy_ with 3 fractions (RT_2 Gy × 3 f’x_) and (D) RT_9 Gy_ with 3 fractions (RT_9Gy × 3 f’x_); and sequential groups treated with regorafenib 24 h (p.o., q.d. _×_ 3 d) after (C) RT_2 Gy × 3 f’x_ and (E) RT_9 Gy × 3 f’x_. (Figure 1A,B).

#### 2.2.4. Sample Preparation

A 150 µL blood sample was withdrawn from the jugular vein with a fraction collector at 0.25, 0.5, 0.75, 1, 1.5, 2, 2.5, 3, 3.5 and 4 h, following drug administration. The samples were centrifuged for 10 min, at 4200× *g*. Then, 50 µL of the resulting plasma was vortexed with 1 mL of ethyl acetate and centrifuged at 5900× *g*. The upper layer was transferred to a new tube and evaporated to dryness.

#### 2.2.5. High-Performance Liquid Chromatography–Ultraviolet (HPLC–UV)

The HPLC system included chromatographic pumps (LC-20AT; Shimadzu Co., Kyoto, Japan), an autosampler (SIL-20AC; Shimadzu Co., Kyoto, Japan) and a UV–Vis detector (SPDM20A; Shimadzu Co., Kyoto, Japan). A Waters Acquity C^18^ column (50 × 2.1 mm, particle size 1.7 μm, Eclipse XDB, Agilent, Palo Alto, CA, USA) was used for sample analysis. The mobile phase consisted of potassium dihydrogen phosphate (10 mM, pH = 3) and acetonitrile (55:45, *v*/*v*). The flow rate was set to 0.2 mL/min, and the injection volume was 5 μL. The temperature in the autosampler was set to 40 °C. The UV–Vis detector scanned from 190 to 300 nm, and the chromatographic profiles were monitored at 265 nm for regorafenib and diethylstilbestrol (internal standard (IS)).

#### 2.2.6. Regorafenib Plasma Extraction

The process for sample extraction was as follows: 50 μL of rat plasma was mixed with internal standard (10 μL, IS, diethylstilbestrol) solution and methanol (140 μL) for protein precipitation. The samples were vortexed and centrifuged at 13,000× *g*, at 4 °C. The supernatants were purified with a filter before HPLC–UV analysis.

#### 2.2.7. Calibration Curves

The calibration curves covered a concentration range from 0.1 to 50 μg/mL. The coefficient of determination (*r*^2^) was used to check the linearity of the assay and was greater than 0.995. The limit of detection (LOD) was the concentration that generates a signal-to-noise ratio of 3. The lower limit of quantification (LLOQ) was the lowest concentration of the linear regression with a signal-to-noise ratio of 10. The 0.01 mg/mL limit of quantification was defined as the lowest concentration on the calibration curve that could be measured routinely with acceptable bias and relative SD.

#### 2.2.8. Accuracy and Precision Evaluation

The bias (%) = (observed concentration—nominal concentration) × 100/nominal concentration was defined as the accuracy. The relative standard deviation, RSD% = (SD) × 100/observed concentration, was defined as the precision. Calibrations in six replicates on the same day (intraday) and on six successive days (interday) were performed to verify the accuracy and precision. Regorafenib was prepared at concentrations of 0.1, 0.5, 1, 5, 10 and 50 μg/mL. The calibration curve was described, using the peak area ratio of regorafenib hydrochloride versus the concentration.

#### 2.2.9. Organ Distribution

Organs, including the brain, liver, heart, spleen, lung and kidney, were collected, weighed and stored at −20 °C, until analysis.

#### 2.2.10. Organ Samples

The organ samples were homogenized in 50% aqueous acetonitrile (sample weight: volume = 1:5) and centrifuged at 13,000× *g*, for 10 min, at 4 °C. The supernatant was stored at −20 °C, until analysis. Additionally, 150 μL of IS solution (diethylstilbestrol) was combined with each organ sample (50 μL) for protein precipitation. Finally, the filtrate (20 μL) was analyzed by HPLC.

#### 2.2.11. Hepatic and Renal Functions

Glutamic-pyruvic transaminase (GPT) and creatine were measured to check the influence of different modalities on hepatic function and renal function by a standard colorimetric method, using a Synchron LX20 spectrophotometer (Beckman Coulter) and manufacturer-supplied reagents.

#### 2.2.12. Pharmacokinetics and Data Analysis

Pharmacokinetic parameters, including the area under the concentration versus time curve (AUC), the clearance (CL), the elimination half-life (*t*_1/2_), the volume of distribution at steady state (Vss) and the mean residence time (MRT), were calculated, using the pharmacokinetics calculation software WinNonlin Standard Edition, Version 1.1 (Scientific Consulting, Apex, NC, USA), by a compartmental method.

### 2.3. In Vitro Study

#### 2.3.1. Cell Viability Assay

Human hepatoma Huh-7 and Hep G2 cell lines kindly provided by Professor Hu (Taipei Veterans General Hospital, Taiwan) were plated into 96-well plates (1 × 10^3^ per well) with serum-containing medium (100 μL) for 1 day. Regorafenib at concentrations of 0, 5, 10 and 20 μmol/L (μM) was added to the plates. Then, the study groups were designed: the concurrent group (1 h after irradiation) or sequential group (24 h after irradiation) with sham RT (RT_0 Gy_), 2 Gy (RT_2 Gy_) and 9 Gy (RT_9 Gy_). Additionally, after 24 h, 20 μL of 5 mg/mL MTT was added to the plates and incubated for 3 h. The supernatant was discarded, the precipitate was dissolved in 200 μL DMSO, and the plates were read with a microplate reader at 570 nm and a reference wavelength of 630 nm.

#### 2.3.2. Morphological Observation

Huh-7 cells were treated with regorafenib at concentrations of 0, 5, 10 and 20 μmol/L (μM) in the concurrent and sequential groups with different RT doses. Then, the cells were centrifuged by using Cytospin (Shandon Inc., Pittsburgh, PA). Liu’s A solution and Liu’s B solution were used on glass slides for 45 and 90 s, respectively. Then, a light microscope (Olympus, Tokyo, Japan) was used to observe the Huh-7 cells.

#### 2.3.3. Cell Cycle Analysis

The cells were collected, fixed and stained with propidium iodide. Flow cytometry was performed, using a Beckman Coulter Elite Epics sorter. The sub-G1 phase was used to quantify dead cells in the apoptosis assays. Cells were released into DMSO or regorafenib at 5, 10 and 20 μM in the concurrent or sequential groups with different RT doses. Floating and adherent cells were harvested at various time points, stained with propidium iodide and analyzed by flow cytometry.

#### 2.3.4. Apoptosis Assay

Annexin-V FITC and propidium iodide (PI) (BD Bioscience Pharmingen (San Diego, CA, USA)) were used to identify apoptotic cells by FACScan. Cells that were positive for Annexin V and negative for PI were defined as early apoptotic cells. Cells that were positive for both Annexin V and PI were defined as late apoptotic cells.

#### 2.3.5. Colony Formation Assays

HA22T cells kindly provided from professor Hu (Taipei Veterans General Hospital, Taiwan) were treated with trypsin to detach, counted and plated (400 per plate) into dishes measuring 60 mm, with either 0.05% DMSO or 16 μmol/L regorafenib with or without irradiation and allowed to grow for 10 days. Cells were stained, and colonies containing ≥ 50 cells were counted.

### 2.4. Calculations and Data Analysis

All statistical calculations were performed with Statistical Product and Service Solutions (SPSS) for Windows, version 20.0 (SPSS, IBM, Armonk, NY, USA). All data are expressed as the mean ± standard deviation (SD). One-way ANOVA with Dunn’s post hoc test was used for comparisons between groups, and statistically significant differences were defined as * *p* < 0.05 or ** *p* < 0.01.

## 3. Results

### 3.1. Results of Pharmacokinetics for Regorafenib with or without Radiotherapy

#### 3.1.1. Optimization of HPLC–UV Conditions

**Figure 2:**
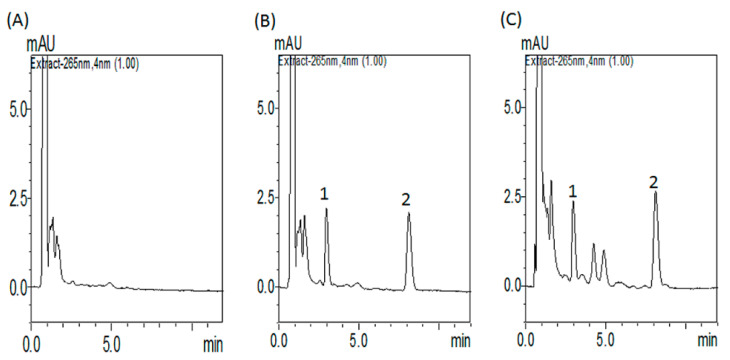
HPLC–UV chromatograms of (**A**) blank plasma samples; (**B**) blank plasma samples spiked with regorafenib (1 µg/mL) and internal standard (IS, 0.8 µg/mL); and (**C**) regorafenib (1.5 µg/mL) and internal standard (IS) (0.8 µg/mL) collected 180 min after regorafenib (16 mg/kg, p.o.) administration alone. Peak 1: internal standard, diethylstilbestrol. Peak 2: regorafenib. The retention time of regorafenib was 8.1 min, with good separation and no endogenous interference in the rat plasma samples, and the procedure exhibited good selectivity.

The mobile phase of 45% ACN and 55% 10 mM KH_2_PO_4_ (*v*/*v*) (pH 3.0) with a Waters ACQUITY BEH C18 column (1.7 µm, 50 × 2.1 mm) produced acceptable separation of regorafenib in the experiment. The retention time of regorafenib was 8.1 min, with good separation and no endogenous interference in the rat plasma samples, and the procedure exhibited good selectivity (Figure 2A–C). Good linearity was achieved in the range of 0.1–50 μg/mL, with all coefficients of correlation greater than 0.995.

#### 3.1.2. Method of Validation of Linearity, Recovery, Precision, Accuracy and Stability

In the current study, the LOD of regorafenib in the plasma was 0.5 μg/mL. The regression equation for regorafenib was y = 1.3824x − 0.0438 (r^2^ = 0.9996) in rat plasma (Supplementary Figure S1). The intraday accuracy of regorafenib ranged from −5.40 to 5.62%. The intraday precision ranged from 0.32 to 14.3%. The interday accuracy ranged from −0.67 to 8.65%. The interday precision ranged from 0.16 to 7.23%. The intraday and interday precision and accuracy values of regorafenib in the plasma were within 15% (Supplementary Table S1)

#### 3.1.3. Both RT_2Gy_ and RT_9Gy_ Modulated the Area under the Concentration Versus Time Curve (AUC) of Regorafenib in the Plasma of Freely Moving Rats

**Figure 3:**
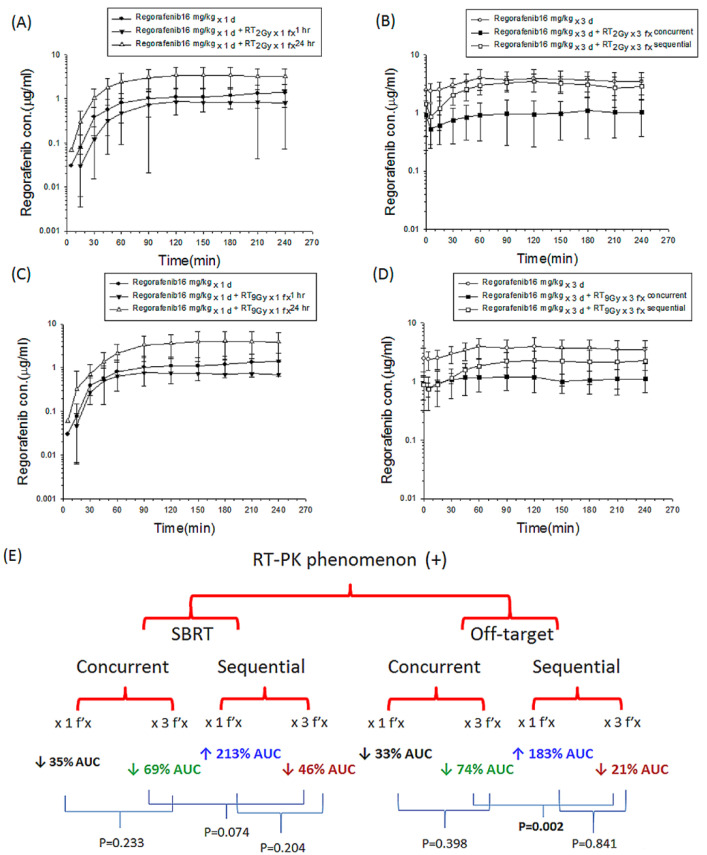
The concentration versus time curves of regorafenib in the plasma of rats obtained for different time courses with or without irradiation (RT). (**A**) The one fraction groups included a sham group, regorafenib with RT_0 Gy_ (regorafenib × 1 d); a concurrent group treated with regorafenib 1 h after RT_2 Gy_ with 1 fraction (RT_2 Gy × 1 f’x_); and a sequential group treated with regorafenib 24 h after RT_2 Gy × 1 f’x_. (**B**) The multiple fraction treated groups included a sham group, regorafenib (p.o., q.d. × 3 d) with RT_0 Gy_ (regorafenib × 3 d); a concurrent group treated with regorafenib (p.o., q.d. × 3 d) 1 h after RT_2 Gy_ with 3 fractions (RT_2 Gy × 3 f’x_); and a sequential group treated with regorafenib 24 h (p.o., q.d. × 3 d) after RT_2 Gy × 3 f’x_. (**C**) The one fraction treatment group included a sham group, regorafenib × 1d; a concurrent group treated with regorafenib 1 h after RT_9 Gy × 1 f’x_ and a sequential group treated with regorafenib 24 h after RT_9 Gy × 1 f’x_. (**D**) The multiple fraction groups included a sham group, regorafenib × 3 d; a concurrent group treated with regorafenib (p.o., q.d. × 3 d) 1 h after RT_9 Gy × 3 f’x_; and a sequential group treated with regorafenib 24 h (p.o., q.d. × 3 d) after RT_9 Gy × 3 f’x_. (**E**) The changes in the area under the concentration versus time curve (AUC) of regorafenib with or without RT. Data are expressed as the mean ± SEM (*n* = 6 for each group).

Radiation at 2 Gy was the daily treatment dose for a human, and the off-target dose was considered to be the dose received around the target that received an ablation RT dose. RT 9 Gy simulated the SBRT dose in clinical practice. In the concurrent RT_2 Gy_ regimen with one fraction (RT_2 Gy × 1 f’x_), the plasma AUC of regorafenib (AUC_regorafenib_) decreased by 33.0% (*p* = 0.356) compared to that of the sham RT group (Figure 3A). Similarly, the AUC_regorafenib_ decreased by 74.0% (*p* = 0.001) in the concurrent RT_2 Gy × 3 f’x_ group compared to the regorafenib _× 3 d_ group (Figure 3B). Interestingly, there were no significant differences in AUC_regorafenib_ between the concurrent RT_2 Gy × 1 f’x_ and RT_2 Gy × 3 f’x_ groups (Table 1).

In contrast to the concurrent regimen, the AUC_regorafenib_ was increased by 182.8% in the sequential RT_2 Gy × 1 f’x_ group (*p* = 0.011) compared to the regorafenib _× 1 d_ group (Figure 3A). Nevertheless, RT decreased the AUC_regorafenib_ by 20.7% in the sequential RT_2 Gy × 3 f’x_ group compared with the regorafenib _× 3 d_ group (*p* = 0.336) (Figure 3B). There was no difference between the sequential RT_2 Gy × 1 f’x_ and RT_2 Gy × 3 f’x_ groups. In other words, the role of fractionation for RT_2 Gy_ in modulating AUC_regorafenib_ is limited regardless of concurrent or sequential regimen. The AUC_regorafenib_ in the sequential RT_2 Gy × 3 f’x_ group was 2.0-fold as much as that in the concurrent RT_2 Gy × 3 f’x_ group (Table 1).

Intriguingly, the AUC_regorafenib_ was increased by 213.2% in the sequential RT_9 Gy × 1 f’x_ group compared with the regorafenib _× 1 d_ group (*p* = 0.016) (Figure 3C). There was no statistically significant difference in AUC_regorafenib_ between the regorafenib _× 1 d_ and concurrent RT_9 Gy × 1 f’x_ groups (Figure 3C). Moreover, there was no statistically significant difference in AUC_regorafenib_ between the concurrent RT_9 Gy × 1 f’x_ and RT_9 Gy × 3 f’x_ groups. Compared to the regorafenib _× 3 d_ group, the AUC_regorafenib_ decreased by 69.4% in the concurrent RT_9 Gy × 3 f’x_ group (*p* = 0.001) and by 45.8% in the sequential RT_9 Gy × 3 f’x_ group (*p* = 0.034). The AUC_regorafenib_ for the sequential RT_9 Gy × 3 f’x_ group was 77.3% higher than that for the concurrent RT_9 Gy × 3 f’x_ group (*p* = 0.074) (Figure 3D and Table 1). The concurrent multiple fractionations of RT decreased AUC_regorafenib_ by approximately 70% at both 2 Gy and 9 Gy, and the Cl values in the concurrent RT_2 Gy × 3 f’x_ and RT_9 Gy × 3 f’x_ groups were higher than in the regorafenib _× 3 d_ group. Combining these observations suggests that the concurrent regimen might facilitate the elimination of regorafenib. Additionally, one shot of RT, whether at 2 or 9 Gy, followed by regorafenib clearly increased the AUC_regorafenib_ (Figure 3E).

The Vss values in the concurrent RT_2 Gy × 3 f’x_ and RT_9 Gy × 3 f’x_ groups were larger than in regorafenib _× 3 d_. Additionally, the Vss in the sequential RT_9 Gy × 3 f’x_ group was larger than that in regorafenib _× 3 d_. This result suggested that both the concurrent and sequential SBRT groups had smaller fluctuations than the regorafenib-only group.

#### 3.1.4. Organ Distributions under Different Regimens of RT and Regorafenib

**Figure 4:**
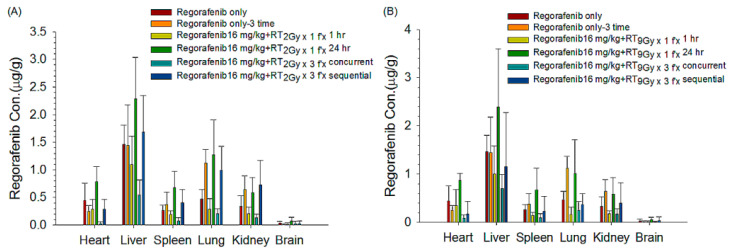
The concentrations (µg/g) of regorafenib in different organs were collected after oral administration for 4 h. The regimens included (**A**) RT_2 Gy × 1 f’x_ and RT_2 Gy × 3 f’x_ or (**B**) RT_9Gy × 1 f’x_ and RT_9 Gy__× 3 f’x_, with or without regorafenib, at a dose of 16 mg/kg, concurrently or sequentially. Data are expressed as the mean ± SEM (*n* = 6 for each group).

Both the concurrent RT_2 Gy__× 3 f’x_ and RT_9 Gy__× 3 f’x_ regimens obviously decreased the biodistribution of regorafenib in the heart, liver, lung, spleen and kidneys compared to that in the control (regorafenib _× 3 d_) group. The sequential RT_2 Gy__× 1 f’x_ and RT_9 Gy__× 1 f’x_ regimens increased the biodistribution of regorafenib in the heart, liver, lung, spleen and kidneys compared to the control (regorafenib _× 1 d_) group. The concentrations of regorafenib in the brain were very limited (Figure 4A,B, Table 2).

#### 3.1.5. Liver and Renal Functions in Different Regimens of RT and Regorafenib

The liver and renal functions in different regimens of RT and regorafenib were evaluated at 0, 120 and 240 min. The value of glutamate pyruvate transaminase (UI) at 240 min for the regorafenib-only, concurrent RT_2 Gy__× 1 f’x_, sequential RT_2 Gy__× 1 f’x_, concurrent RT_9 Gy__× 1 f’x_ and sequential RT_9 Gy__× 1 f’x_ groups were 36.3 ± 15.0, 31.8 ± 5.3, 30.5 ± 6.0, 33.5 ± 8.8 and 33.7 ± 5.2, respectively. Additionally, the values of creatinine (mg/dL) at 240 min were 0.23 ± 0.04, 0.25 ± 0.06, 0.22 ± 0.04, 0.21 ± 0.02 and 0.25 ± 0.04, respectively. There were no significant differences in glutamate pyruvate transaminase and creatinine between regorafenib with or without RT in the different regimens and doses.

### 3.2. In Vitro Study

#### 3.2.1. Cell Viability Analysis

**Figure 5:**
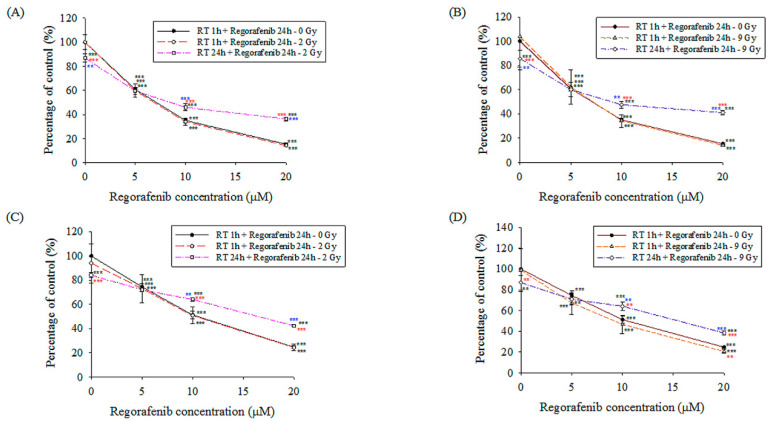
Huh-7 and Hep G2 cells were seeded into 96-well plates (1 × 10^3^ per well) in 100 μL of serum-containing medium and allowed to grow for 1 day. Concentrations of 0, 5, 10 and 20 μmol/L (μM) regorafenib were added to the plates, 1 h following irradiation (concurrent group) or 24 h following irradiation (sequential group), with sham RT (RT_0 Gy_), 2 Gy (RT_2 Gy_) and 9 Gy (RT_9 Gy_). (**A**) Huh-7 cells treated with RT_2 Gy_; (**B**) Huh-7 cells treated with RT_9 Gy_; (**C**) Hep G2 cells treated with RT_2 Gy_; (**D**) Hep G2 cells treated with RT_9 Gy_. Data from three separate experiments are expressed as the mean ± standard error of the mean (SEM). Black asterisk (*): all vs. 0 Gy + 0 mM. Red asterisk (*): RT 1 h + regorafenib 24 h or RT 24 h + regorafenib 24 h vs. 0 Gy in different concentrations. Blue asterisk (*): RT 1 h + regorafenib 24 h vs. RT 24 h + regorafenib 24 h. ** *p* ˂ 0.01, *** *p* ˂ 0.001.

The regorafenib concentrations studied ranged from 0 to 20 μM, and the estimated concentration at which 50% of cells were killed (IC50) for Huh-7 and Hep G2 were shown in the Table 3. The viability of Huh-7 and Hep G2 cells treated with 0, 5, 10 and 20 μM regorafenib only was 100% and 100%, 60.9 ± 1.8% and 74.6 ± 2.3%, 35.1 ± 1.6% and 51.3 ± 3.1%, 15.4 ± 1.0% and 24.7 ± 1.2%, respectively (Figure 5). However, there were no synergistic effects of the concurrent administration of regorafenib with the RT_2 Gy_ and RT_9 Gy_ regimens, when compared with regorafenib only. Interestingly, the viability of Huh-7 and Hep G2 cells with regorafenib (10 and 20 μM) following RT_2 Gy_ and RT_9 Gy_ treatment was higher than the regorafenib-only and concurrent regimen. However, there were no differences at regorafenib 5 μM between regorafenib-only, concurrent and sequential regimens.

#### 3.2.2. Morphological Changes

Cell shrinkage and pyknosis occurred in a dose-dependent manner with respect to regorafenib and RT. Additionally, the cell outlines were irregular with condensed and peripheralized chromatin. Significant numbers of apoptotic bodies were observed in the sequential regimen of RT_9 Gy_ with dose dependence on regorafenib. In contrast, the cytoplasmic vacuoles were more prominent in the concurrent regimen, showing dose dependence on regorafenib at both RT doses. Moreover, cell swelling, the formation of cytoplasmic vacuoles and cytoplasmic blebs and loss of cell membrane integrity were clearly observed in both the concurrent and sequential regimens of regorafenib (20 μM) with RT (Supplementary Figure S2A,B).

#### 3.2.3. Cell Cycle Analysis

**Figure 6:**
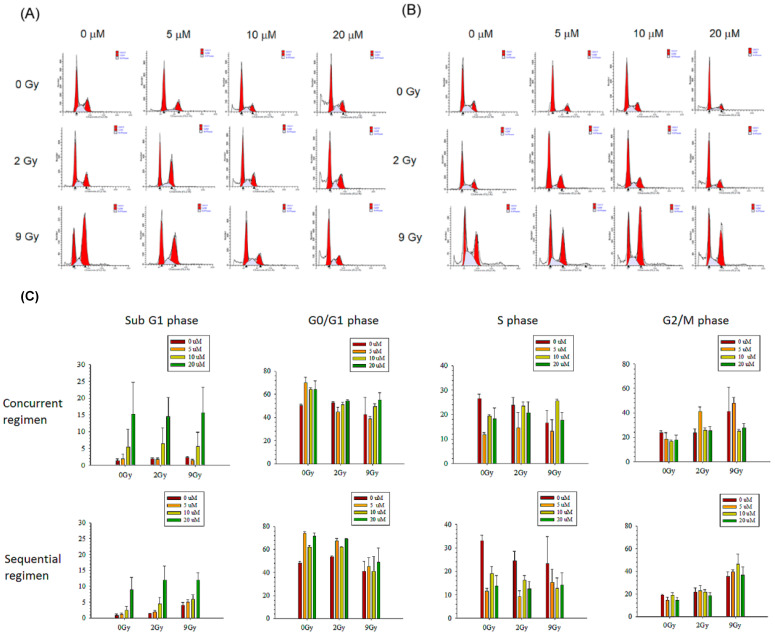
Huh-7 cells were treated with regorafenib at concentrations of 0, 5, 10 or 20 μmol/L (μM). (**A**) The percentage of hypodiploid cells (sub-G1) was quantified in plates 1 h after irradiation (concurrent group) with sham RT (RT_0 Gy_), 2 Gy (RT_2 Gy_) and 9 Gy (RT_9 Gy_). (**B**) The percentage of hypodiploid cells (sub-G1) was quantified in plates 24 h after irradiation (sequential group) with different RT doses. (**C**) Bar graphs showing the cell cycle distributions of the different treatment regimens. Data from three separate experiments are expressed as the mean ± standard error of the mean (SEM).

Huh-7 cells were gated into sub-G1, G1, S and G2/M by flow cytometric cell-cycle analysis. As the histogram shows, the accumulation of sub-G1 Huh-7 cells in the concurrent groups was correlated with the dose of regorafenib but was not correlated with RT or its absence. However, the accumulation of sub-G1 Huh-7 cells in the sequential groups was correlated with the doses of regorafenib and RT (Figure 6A–C).

#### 3.2.4. Apoptosis Analysis

Detection of necrotic and apoptotic cells by Annexin V and PI double-stain labeling. Regorafenib concurrent with or following RT caused Huh-7 cell apoptosis in a dose-dependent manner. There were no obvious synergistic effects of apoptosis in the concurrent regimen (Supplementary Figure S3A). However, RT followed by regorafenib enhanced the late apoptosis of Huh-7 cells. Interestingly, RT_2 Gy_ followed by regorafenib at 20 μM, showed obvious cell-apoptotic phenomena (Supplementary Figure S3B).

#### 3.2.5. Colony Formation Analysis

**Figure 7:**
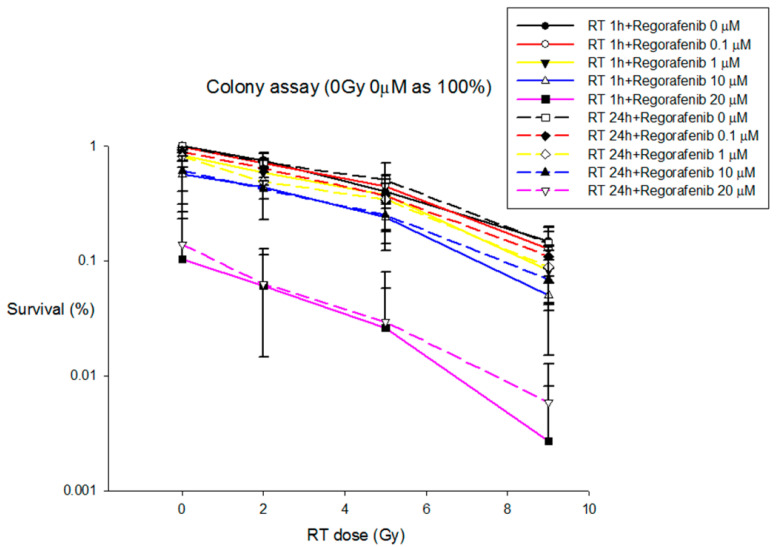
The colony formation analysis for HA22T line that were treated with indicated concentrations (from 0.1 to 20 µM) of regorafenib and RT (2 and 9 Gy), respectively. Data from three separate experiments are expressed as the mean ± standard error of the mean (SEM).

HA22T cancer cells were treated with concurrent and sequential regimen at different concentrations of regorafenib and different doses of RT. The results revealed that colony formation of HA22T were decreased in the presence of the indicated concentrations (from 1 to 20 μM) of regorafenib and RT (2 and 9 Gy), suggesting the inhibitory potential of regorafenib and RT against HA22T cell with dose dependent manners. Additionally, under concentration (1, 10 and 20 μM) of regorafenib, concurrent or sequential with RT had synergic effects to inhibit the colony formation when compared with regorafenib alone. However, there were no differences between concurrent and sequential regimen in RT with different doses level (Figure 7).

## 4. Discussion

Regorafenib is an oral multikinase inhibitor [19] that blocks the activity of multiple protein kinases involved in angiogenesis (vascular endothelial growth factor receptor (VEGFR)-1, -2, -3 and tyrosine kinase with immunoglobulin and epidermal growth factor homology domain 2 (TIE-2, a crucial regulator of angiogenesis)) [20], oncogenesis (c-kit, Raf-1, c-Ret and V600E-mutated B-Raf), metastasis and the tumor microenvironment (platelet-derived growth factor receptor; and fibroblast growth factor receptor) [19]. Regorafenib provides an overall survival benefit in HCC patients with progression on sorafenib treatment [7]. Moreover, the treatment of patients with sorafenib followed by regorafenib resulted in an unprecedented median overall survival of 26 months [21]. Therefore, the NCCN Guidelines^®^ list regorafenib as a systemic therapy for patients with HCC progression on or after sorafenib.

Overactivation of the phosphatidylinositol 3 kinase (PI3K)/AKT and mitogen-activated protein kinase (MAPK) pathways is a well-known trait in cancer, and compounds or modalities that suppress these signaling pathways represent an attractive approach to strengthening the effectiveness of regorafenib. Recently, regorafenib combined with different compounds was reported to exert enhanced antitumor effects compared with the effects of individual administration [22]. Intriguingly, radiation exposure activates the expression of MAPK and PI3K [23], and the phosphorylation levels of phospho-c-Jun N-terminal kinase (JNK), ERK, Akt and p38 are upregulated significantly in irradiated HCC cells [24]. Regorafenib induces both extrinsic and intrinsic apoptotic pathways by suppressing ERK/NF-ĸB activation in SK-HEP-1 cells [25]. These lines of evidence provide the rationale for the combination of regorafenib and irradiation.

Cancer treatment with a VEGF inhibitor has the potential to result in acquired resistance [26] and rapid vascular regrowth after removal of the anti-VEGF therapy [27]. The NCCN Guidelines^®^ also suggest that EBRT and SBRT could be applied in patients with unresectable or inoperable HCC. Irradiation induces hypoxia and VEGF upregulation, which are related to radioresistance [28]. In the current study, the Huh-7 and Hep G2 cells treated with 10 and 20 μM of regorafenib showed higher viability in the sequential regime that suggested the possibility of radioresistance in the sequential regime. By inhibiting the activity of VEGF, receptor blockade agents can potentially act as radiosensitizers. Nuclear factor kappa B (NF-*κ*B) is a well-defined radiation-responsive transcription factor [29]. Notably, NF-*κ*B also modulates the expression of CYP3A4 [30] and increases the expression of VEGF [31]. Regorafenib is metabolized via CYP3A4 [19]. Additionally, regorafenib [32] selectively inhibits the radiation-induced activation of VEGFR and thereby enhances the effectiveness of irradiation possibly. Interestingly, the Huh-7 and Hep G2 cells treated with 5 μM of regorafenib, there were no significant differences between drug only, concurrent and sequential regimens although higher viabilities with 10 and 20 μM of regorafenib were noted in the sequential regimes. Additionally, regorafenib concurrent or sequential with RT had synergic effects to inhibit the colony formation when compared with regorafenib alone. Moreover, RT_9 Gy_ was more efficient to inhibit colony formation than RT_2 Gy._ Consequently, the potential for a synergistic anti-angiogenesis effect makes the combination of diaryl urea agents and RT theoretically attractive.

Nevertheless, the optimal timing, duration, and dosing of regorafenib when used in combination with RT or SBRT remain unknown. Roberto et al. [15] reported a man affected by metastatic colorectal cancer (mCRC) who was treated with regorafenib combined with multiple fractions of SBRT with 54 Gy, achieving a durable response without severe toxicities. Gatto et al. [33] also reported a patient affected by metastatic gastrointestinal stromal tumor who was treated with RT (34 Gy in 14 fractions) combined with regorafenib (160 mg/day), achieving an objective response. In the current study, the morphology data showed apoptosis of Huh-7 cells in the concurrent regimen with dose dependence of regorafenib at both RT doses. However, apoptosis analysis did not clearly show synergistic effects of the concurrent regimen on apoptosis in Huh-7 cells. The concurrent regimen decreased the AUC_regorafenib_, regardless of the off-target dose or SBRT dose. Multiple fractions and a single fraction of RT decreased regorafenib levels by approximately 70% and 35%, respectively. There were no significant differences in AUC_regorafenib_ between one fraction and multiple fractions of 2 Gy in the concurrent regimen. The concurrent RT_9Gy_ regimen showed a similar trend. Considering the irradiated-volume effect, SBRT is more efficacious than conventional techniques for modulating the PK of regorafenib. Additionally, a low-dose bath caused by arc therapy can modulate the PK of regorafenib. Furthermore, concomitant RT and regorafenib decreased the AUC_regorafenib_, which may decrease drug-related toxicity.

In contrast, Tian et al. [14] reported an mCRC patient treated with SBRT (20 Gy in a single fraction) followed by regorafenib who developed hyperalgesia and radicular pain. RT upregulated the phosphorylation levels of phospho-c-JNK in HCC cells [24]. Additionally, JNK and its target phospho-c-Jun were upregulated at 24 and 48 h in Hep3B cells after regorafenib treatment [34]. Interestingly, in the current study, cell morphology, cell cycle and apoptosis analyses showed that the sequential regimen resulted in synergistic and dose-dependent effects on apoptosis in Huh-7 cells. Additionally, compared to regorafenib only, the concurrent regimen did not increase the inhibition of viability of Huh-7 and Hep G2 cells. Moreover, a single fraction of RT followed by regorafenib increased AUC_regorafenib_ approximately twofold, which might partially explain Tian’s report [14]. Additionally, the AUC_regorafenib_ in the sequential RT_2 Gy__× 3 f’x_ group was 2.2-fold higher than that in the concurrent RT_2 Gy__× 3 f’x_ group. Similarly, the sequential RT_9 Gy__× 3 f’x_ regimen increased AUC_regorafenib_ by 69%compared with the concurrent regimen. It is apparent that the synergistic effect of inhibitor treatment and irradiation and the PK modulation of regorafenib support the use of a sequential rather than a concurrent regimen.

The daily interaction between RT and regorafenib is much less well understood. In the concurrent regimen, the next daily dose of regorafenib becomes a sequential dose relative to the previous RT. There was no statistically significant difference between sequential and concurrent regimens in multiple fractions of SBRT. In other words, the influence of regimen in the SBRT technique with regorafenib is limited. In contrast, the sequential RT_2 Gy__× 3 f’x_ regimen increased AUC_regorafenib_ 2.2-fold compared with that in the concurrent regimen. The C_max_ of regorafenib was decreased in the concurrent RT_2 Gy__× 3 f’x_ and RT_9 Gy__× 3 f’x_ groups. Additionally, the Cl of regorafenib was increased in the concurrent RT_2 Gy__× 3 f’x_ and RT_9 Gy x 3 f’x_ groups. A decreased C_max_ and increased Cl during the concurrent regimen indicated that both off-target and SBRT doses in local liver RT reduce the absorption of regorafenib and increase its elimination. In other words, the sequential regimen resulted in a greater impact of regorafenib than the concurrent regimen.

Several studies have shown that the addition of RT to diaryl urea agents is well tolerated [9,35,36,37]. However, increased toxicity has been reported with the combination of RT and VEGF inhibitors [13,14,38,39]. Our previous study demonstrated that the off-target radiation dose significantly modulated the bioavailability of chemotherapy agents [16,40,41]. Advanced RT techniques allow the delivery of large doses of radiation; nonetheless, areas other than the target area are exposed to significant low-dose radiation [16]. Therefore, the 2 Gy dose used in the current study could also be viewed as an off-target dose during SBRT, although 2 Gy is a daily conventional dose. The current study noted that both off-target and SBRT doses could modulate the AUC of regorafenib in sequential design. For that reason, advanced radiotherapy combined with regorafenib should consider the low-dose “bath” effects [42], especially in the sequential regimen.

The current data suggested that the organ distributions for organs at risk (heart, spleen, lung, kidney and lightly brain) were enhanced and the AUC of regorafenib was increased in the single fraction sequential regimen. The organ distributions were decreased in the multiple fraction concurrent regimen. Additionally, the distribution of regorafenib in the brain was detected and upregulated in the sequential regimen even though the concentration in the brain was low. Recently, a similar observation that regorafenib and its metabolites could be detected in patients’ cerebrospinal fluid has been reported [43], which supports the current results. However, grade-three or above adverse effects have been reported in patients treated with regorafenib, including heart failure, hypertension, thrombocytopenia, hyperbilirubinemia, increased aspartate aminotransferase and alanine aminotransferase, and gastrointestinal toxicities [7,44,45,46]. In the development of new radiation-modulated strategies and the design of clinical trials, the unexplained biological enhancements of the effects of regorafenib by the RT–PK phenomenon should be addressed cautiously to avoid severe toxicity when RT and regorafenib are used as synergistic tools in cancer treatment strategies.

This study had some limitations. First, the current study was designed to examine the interaction between RT and the PK of regorafenib but did not include the pharmacodynamics of regorafenib during RT. Therefore, the current study cannot describe the treatment effects of the combination of RT and regorafenib, even though the AUC_plasma_ of regorafenib was increased by approximately 2-fold at RT in one fraction sequential regimen and decreased by 70% at RT in the multiple fraction concurrent regimen. Second, the possible mechanism was not addressed in the current study because the presence or absence of the RT–PK phenomenon in the regorafenib plus RT setting could not be ensured before the study. However, we confirmed that the systemic PK of regorafenib could be modulated by either an off-target or SBRT dose of RT. Finally, our initial finding is that RT modulated the clinical efficacy of systemic drugs including chemotherapeutics and targeted therapeutics. To prove this concept, we performed in vivo experiments for PK analysis of regorafenib and found the differential activities between concurrent RT and sequential RT as well as conventional RT and SBRT. The design of these combinatory regimens could truly reflect the different scenarios in clinical practice. We realized that this in vitro study could not represent or validate the in vivo study, due to the lack of impacts of local microenvironment and systemic modulators. In this way, the concurrent and sequential combination of regorafenib and RT induced differential effects on hypoploid (sub-G1 or apoptosis-like) cell populations, post-RT G2/M phase distribution (mitotic arrest population underway DNA damage repair process) and morphological alterations. Therefore, the data of an in vitro study would be regarded as the preliminary work to prove that the differential effects of various RT on PK of regorafenib may have similar correlations in a cellular basis. To determine the possible mechanism and effects of regorafenib administered concurrently or sequentially with RT, further studies are clearly required, including more HCC cell lines of colony formation assays and DNA double-strand break (DSB) analysis by staining for γH2AX and/or 53BP1, to examine the effects of single and double treatment on clonogenic survival and DNA repair in additional hepatocarcinoma cell lines, in vivo tumor models, or clinical trials.

## 5. Conclusions

To our knowledge, our study is the first to show the radiation–drug interaction between RT and regorafenib. EBRT and SBRT doses possess a similar ability to modulate the AUC of regorafenib in systemic therapy. The concurrent or sequential regimen for regorafenib with EBRT and SBRT may influence the AUC and biodistribution during treatment and may be correlated with the effects and toxicities. The current data provide insight into the possibility that a sequential regimen be a more efficient schedule than a concurrent regimen for achieving a synergistic effect of regorafenib and irradiation. However, the unexplained biological enhancements of the effects of regorafenib by RT for organs at risk need to be carefully observed in the daily practice though the concentration in the brain is increasing lightly. Together, these data support the RT–PK phenomenon in our study, and the impact in the sequential regimen might be more obviously than concurrent regimen. The studies of pharmacodynamics and clinical trials to confirm the applications of RT–PK phenomenon of regorafenib are warranted in the future.

## Figures and Tables

**Table 1:** Pharmacokinetic parameters of regorafenib (16 mg/kg, p.o.) with and without RT 2 and 9 Gy.

Parameter	AUC_0-T_	C_max_	T_max_	t_½_	Cl	Vss	MRT
Unit	min mg/mL	mg/mL	min	min	mL/min/kg	mL/kg	min
Regorafenib 16 mg/kg × 1 d	226.8 ± 122.7	1.45 ± 0.77	190 ± 68	942.3 ± 535.1	8.18 ± 3.06	12641.9 ± 6928.9	1483 ± 1066
Regorafenib 16 mg/kg × 3d	849.6 ± 317.3 ^a^	4.19 ± 1.53	90 ± 63	550 ± 146	4.21 ± 1.82	3604.5 ± 951.1	823 ± 341
RT_2 Gy × 1 f’x_ with regorafenib 16 mg/kg × 1d	152.2 ± 143.5	0.92 ± 0.85	165 ± 59	409 ± 150	16.6 ± 7.21	21606.9 ± 20731.8	651 ± 215
RT_2 Gy × 1 f’x_ followed by regorafenib 16 mg/kg × 1 d	641.8 ± 305.1 ^b,e^	3.63 ± 1.76	180 ± 63	346 ± 93	6.19 ± 0.35	3081.4 ± 773.1	553 ± 116
RT_2 Gy × 3 f’x_ with regorafenib 16 mg/kg × 3 d	223.0 ± 134.0 ^d,j^	1.24 ± 0.79^l^	125 ± 58	372 ± 152	23.20 ± 9.45 ^n^	12759.6 ± 8467.4 ^p^	584 ± 186
RT_2 Gy × 3 f’x_ followed by regorafenib 16 mg/kg × 3 d	673.7 ± 224.1 ^c,f^	3.55 ± 1.08	100 ± 24	920 ± 956	8.56 ± 7.21	4156.0 ± 1959.9	1370 ± 1365
RT_9 Gy × 1 f’x_ with regorafenib 16 mg/kg × 1 d	147.5 ± 187.0	0.83 ± 0.99	155 ± 84	654 ± 445	34.6 ± 25.3	30999.0 ± 34618.0	976.3 ± 639.4
RT_9 Gy × 1 f’x_ followed by regorafenib 16 mg/kg × 1 d	711.4 ± 392.8 ^g,k^	4.32 ± 2.71	155 ± 29	608 ± 210	3.3 ± 4.3	2898.8 ± 4123.7	935.1 ± 320.9
RT_9 Gy × 3 f’x_ with regorafenib 16 mg/kg × 3 d	260.0 ± 110.7 ^i^	1.39 ± 0.53 ^m^	98 ± 81	662 ± 598	19.0 ± 11.7 ^o^	11564.4 ± 3361.0 ^q^	983.1 ± 857.7
RT_9 Gy × 3 f’x_ followed by regorafenib 16 mg/kg × 3 d	460.6 ± 220.5 ^h,j^	2.49 ± 1.49	110 ± 78	571 ± 323	11.4 ± 4.5	7960.3 ± 3023.5 ^r^	860.7 ± 467.9

^a^ Regorafenib × 1 d vs. regorafenib × 3 d, *p* = 0.001. ^b^ RT_2 Gy × 1 f’x_ followed by regorafenib vs. regorafenib × 1 d, *p* = 0.011. ^c^ RT_2 Gy × 3 f’x_ followed by regorafenib _×_ 3 d vs. regorafenib × 1 d, *p* = 0.002. ^d^ RT_2 Gy × 3 f’x_ concurrent with regorafenib × 3 d vs. regorafenib × 3 d, *p* = 0.001. ^e^ RT_2 Gy × 1 f’x_ concurrent with regorafenib × 1 d vs. RT_2 Gy x 1 f’x_ followed by regorafenib × 1 d, *p* = 0.005. ^f^ RT_2 Gy × 3 f’x_ concurrent with regorafenib × 3 d vs. RT_2 Gy × 3 f’x_ followed by regorafenib × 3 d, *p* = 0.002. ^g^ RT_9 Gy × 1 f’x_ followed by regorafenib × 1 d vs. regorafenib × 1 d, *p* = 0.016. ^h^ RT_9 Gy × 1 f’x_ followed by regorafenib × 3 d vs. regorafenib × 1 d, *p* = 0.047. ^i^ RT_9 Gy × 3 f’x_ concurrent with regorafenib × 3 d vs. regorafenib × 3 d, *p* = 0.001. ^j^ RT_9 Gy × 3 f’x_ followed by regorafenib × 3 d vs. regorafenib × 3 d, *p* = 0.033. ^k^ RT_9 Gy × 1 f’x_ concurrent with regorafenib × 1 d vs. RT_9Gy × 1 f’x_ followed by regorafenib × 1 d, *p* = 0.010. ^l^ RT_2 Gy × 3 f’x_ concurrent with regorafenib × 3 d vs. regorafenib × 3 d, *p* = 0.002. ^m^ RT_9 Gy × 3 f’x_ concurrent with regorafenib × 3 d vs. regorafenib × 3 d, *p* = 0.001. ^n^ RT_2 Gy × 3 f’x_ concurrent with regorafenib × 3 d vs. regorafenib × 3 d, *p* = 0.01. ^o^ RT_9 Gy × 3 f’x_ concurrent with regorafenib × 3 d vs. regorafenib × 3 d, *p* = 0.05. ^p^ RT_2 Gy × 3 f’x_ concurrent with regorafenib × 3 d vs. regorafenib × 3 d, *p* = 0.03. ^q^ RT_9 Gy × 3 f’x_ concurrent with regorafenib × 3 d vs. regorafenib × 3 d, *p* = 0.001. ^r^ RT_9 Gy × 3 f’x_ followed by regorafenib × 3 d vs. regorafenib × 3 d, *p* = 0.01.

**Table 2:** Concentrations of regorafenib in the heart, liver, spleen, lung, kidney and brain of rats after administration (16 mg/kg, p.o.) with or without radiotherapy.

Organ (µg/g)	Heart	Liver	Spleen	Lung	Kidneys	Brain
Regorafenib 16 mg/kg × 1 d	0.45 ± 0.31	1.46 ± 0.34	0.26 ± 0.10	0.47 ± 0.18	0.34 ± 0.19	0.03 ± 0.04
Regorafenib 16 mg/kg × 3 d	0.25 ± 0.10	1.45 ± 0.74	0.38 ± 0.22	1.12 ± 0.25	0.65 ± 0.25	0.01 ± 0.02
RT_2 Gy × 1 f’x_ with regorafenib 16 mg/kg × 1 d	0.29 ± 0.17	1.10 ± 0.51	0.19 ± 0.07	0.29 ± 0.19	0.21 ± 0.11	0.02 ± 0.03
RT_2 Gy × 1 f’x_ followed by regorafenib 16 mg/kg × 1 d	0.78 ± 0.27 ^a^	2.28 ± 0.75 ^d^	0.68 ± 0.30 ^g^	1.27 ± 0.64 ^l^	0.59 ± 0.27	0.08 ± 0.06
RT_2 Gy × 3 f’x_ with regorafenib 16 mg/kg × 3 d	0.03 ± 0.03 ^b^	0.55 ± 0.27 ^e^	0.08 ± 0.06 ^h^	0.21 ± 0.09 ^m^	0.13 ± 0.06 ^r^	0.02 ± 0.05
RT_2 Gy × 3 f’x_ followed by regorafenib 16 mg/kg × 3 d	0.29 ± 0.18 ^c^	1.68 ± 0.65 ^f^	0.41 ± 0.24 ^i^	0.99 ± 0.43 ^n^	0.73 ± 0.44 ^s^	0.03 ± 0.05
RT_9 Gy × 1 f’x_ with regorafenib 16 mg/kg × 1 d	0.36 ± 0.33	1.01 ± 0.58	0.14 ± 0.07	0.17 ± 0.14 ^o^	0.18 ± 0.05	0.01 ± 0.02
RT_9 Gy × 1 f’x_ followed by regorafenib 16 mg/kg × 1 d	0.87 ± 0.14	2.39 ± 1.20	0.68 ± 0.45 ^j^	1.01 ± 0.70	0.59 ± 0.34	0.06 ± 0.05
RT_9 Gy × 3 f’x_ with regorafenib 16 mg/kg × 3 d	0.09 ± 0.07	0.70 ± 0.29	0.10 ± 0.08 ^k^	0.25 ± 0.18 ^p^	0.17 ± 0.12 ^t^	0.01 ± 0.02
RT_9 Gy × 3 f’x_ followed by regorafenib 16 mg/kg × 3 d	0.17 ± 0.27	1.16 ± 1.12	0.23 ± 0.31	0.37 ± 0.23 ^q^	0.40 ± 0.43	0.04 ± 0.08

^a^ RT_2 Gy × 1 f’x_ concurrent with regorafenib × 1 d vs. RT_2 Gy × 1 f’x_ followed by regorafenib × 1 d, *p* = 0.004. ^b^ RT_2 Gy × 3 f’x_ concurrent with regorafenib × 3 d vs. regorafenib × 3 d, *p* = 0.001. ^c^ RT_2 Gy × 3 f’x_ concurrent with regorafenib × 3 d vs. RT_2 Gy × 3 f’x_ followed by regorafenib × 3 d, *p* = 0.005. ^d^ RT_2 Gy × 1 f’x_ followed by regorafenib vs. regorafenib × 1 d, *p* = 0.036. ^e^ RT_2 Gy × 3 f’x_ concurrent with regorafenib × 3 d vs. regorafenib × 3 d, *p* = 0.018. ^f^ RT_2 Gy × 3 f’x_ concurrent with regorafenib × 3 d vs. RT_2 Gy × 3 f’x_ followed by regorafenib × 3 d, *p* = 0.003. ^g^ RT_2 Gy × 1 f’x_ followed by regorafenib vs. regorafenib × 1 d, *p* = 0.009. ^h^ RT_2 Gy × 3 f’x_ concurrent with regorafenib × 3 d vs. regorafenib × 3 d, *p* = 0.01. ^i^ RT_2 Gy × 3 f’x_ concurrent with regorafenib × 3 d vs. RT_2 Gy × 3 f’x_ followed by regorafenib × 3 d, *p* = 0.008. ^j^ RT_9 Gy × 1 f’x_ concurrent with regorafenib × 1 d vs. RT_9 Gy × 1 f’x_ followed by regorafenib × 1 d, *p* = 0.016. ^k^ RT_9 Gy × 3 f’x_ concurrent with regorafenib × 3 d vs. regorafenib × 3 d, *p* = 0.016. ^l^ RT_2 Gy x 1 f’x_ followed by regorafenib vs. regorafenib × 1 d, *p* = 0.014. ^m^ RT_2 Gy × 3 f’x_ concurrent with regorafenib × 3 d vs. regorafenib × 3 d, *p* = 0.001. ^n^ RT_2 Gy × 3 f’x_ concurrent with regorafenib × 3 d vs. RT_2 Gy × 3 f’x_ followed by regorafenib × 3 d, *p* = 0.001. ^o^ RT_9 Gy × 1 f’x_ concurrent with regorafenib vs. regorafenib × 1 d, *p* = 0.009. ^p^ RT_9 Gy x 3 f’x_ concurrent with regorafenib × 3 d vs. regorafenib × 3 d, *p* = 0.001. ^q^ RT_9 Gy × 3 f’x_ followed by regorafenib × 3 d vs. regorafenib × 3 d, *p* = 0.001. ^r^ RT_9 Gy × 3 f’x_ concurrent with regorafenib × 3 d vs. regorafenib × 3 d, *p* = 0.001. ^s^ RT_2 Gy × 3 f’x_ concurrent with regorafenib × 3 d vs. RT_2 Gy × 3 f’x_ followed by regorafenib × 3 d, *p* = 0.008. ^t^ RT_9 Gy × 3 f’x_ concurrent with regorafenib × 3 d vs. regorafenib × 3 d, *p* = 0.002.

**Table 3:** The regorafenib concentrations studied ranged from 0 to 20 μM concurrent with or following radiotherapy (RT) with sham RT (RT_0 Gy_), 2 Gy (RT_2 Gy_) and 9 Gy (RT_9 Gy_), and the estimated concentration at which 50% of cells were killed (IC50) for Huh-7 and Hep G2.

Regorafenib (μM)	RT_0 Gy_				RT_2 Gy_				RT_9 Gy_			
	C		S		C		S		C		S	
	Huh-7	Hep G2	Huh-7	Hep G2	Huh-7	Hep G2	Huh-7	Hep G2	Huh-7	Hep G2	Huh-7	Hep G2
IC50	6.56	9.87	12.8	17.68	6.38	10.5	12.07	18.89	6.36	8.43	15.23	16.81
0	100.0 ± 0.0^,^	100.0 ± 0.0	100.0 ± 0.0	100.0 ± 0.0	100.1 ± 6.0	94.0 ± 16.2	87.0 ± 3.3	84.3 ± 2.2	103.9 ± 27.4	98.9 ± 20.8	85.7 ± 6.9	86.9 ± 6.9
5	60.9 ± 1.8	74.6 ± 2.4	72.6 ± 1.0	82.0 ± 1.9	59.8 ± 5.5	73.1 ± 11.7	59.5 ± 2.7	72.1 ± 1.7	62.3 ± 14.1	64.7 ± 11.3	60.2 ± 5.6	71.0 ± 5.3
10	35.1 ± 1.6	51.3 ± 3.1	55.2 ± 1.2	67.0 ± 1.3	33.9 ± 2.7	50.8 ± 7.0	46.0 ± 2.9	64.1 ± 1.3	34.3 ± 5.3	46.7 ± 8.9	47.6 ± 3.0	64.2 ± 4.3
20	15.4 ± 1.0	24.7 ± 1.2	41.4 ± 0.4	46.0 ± 3.4	14.4 ± 0.8	24.5 ± 2.5	36.3 ± 1.4	42.4 ± 0.8	14.1 ± 0.7	20.6 ± 1.5	41.1 ± 1.7	38.5 ± 1.9

C: concurrent group, regorafenib was added to the plates 1 h following RT. S: sequential group, regorafenib was added to the plates 24 h following RT.

## Data Availability

The datasets used and/or analyzed are available from the corresponding author on reasonable request.

## Supplementary Materials

The following are available online, at https://www.mdpi.com/1999-4923/13/3/386/s1. Figure S1: Calibration curve for regorafenib in plasma ranging from 0.1 μg/mL to 25 μg/mL. r2: correlation coefficient. Figure S2: Huh-7 cells were treated with regorafenib at concentrations of 0, 5, 10, or 20 μmol/L (μM) either (A) 1 hr after irradiation (concurrent group) or (B) 24 hr after irradiation (sequential group) with sham RT (RT0 Gy), 2 Gy (RT2 Gy) and 9 Gy (RT9 Gy). Figure S3: Huh-7 cells were treated with regorafenib at concentrations of 0, 5, 10, or 20 μmol/L (μM). (A) Cells were stained with Annexin-V FITC and propidium iodide (PI). Annexin V(+)/PI(−) and Annexin V(+)/PI(+) were defined as early and late apoptotic cells, respectively. There were no obvious synergistic effects of apoptosis in the concurrent regimen. (B) RT followed by regorafenib enhanced th. Figure S1: Calibration curve for regorafenib in plasma, Table S1: Interday and intraday assay precision (% RSD) and accuracy (% bias) values for the HPLC-UV determination of regorafenib in rat plasma.
