# Supplementary material for: Effect of Synchronous Versus Sequential Regimens on the Pharmacokinetics and Biodistribution of Regorafenib with Irradiation
Source: Pharmaceutics. 2021 Mar 13;13(3):386. doi: 10.3390/pharmaceutics13030386 (PMC8035703; doi:10.3390/pharmaceutics13030386)
Supplement: Supplementary file 1 [file pharmaceutics-13-00386-s001.pdf]

pharmaceutics

MDPI

Supplementary Materials: Effect of Synchronous Versus Sequential Regimens on the Pharmacokinetics and Biodistribution of Regorafenib with Irradiation

Tung-Hu Tsai, Yu-Jen Chen, Li-Ying Wang and Chen-Hsi Hsieh*

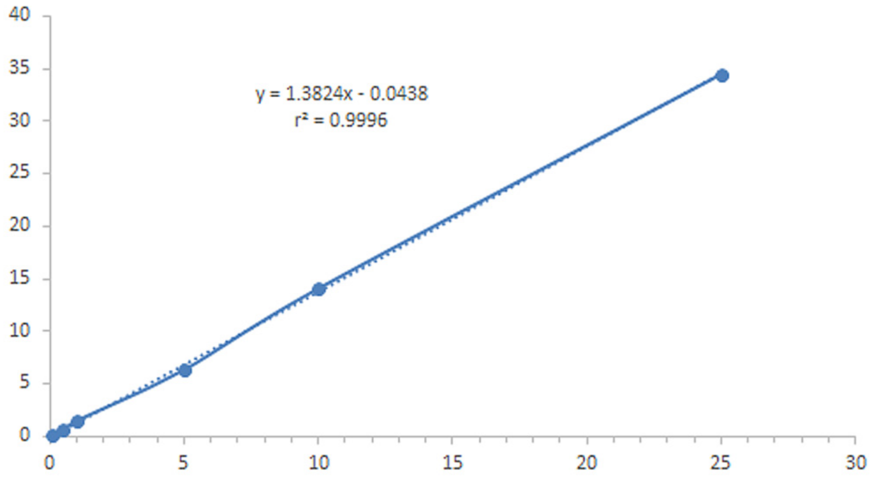

Figure S1. Calibration curve for regorafenib in plasma ranging from 0.1 µg/mL to 25 µg/mL. r2: correlation coefficient.

Publisher's Note: MDPI stays neutral with regard to jurisdictional claims in published maps and institutional affiliations.

Copyright: © 2021 by the authors. Submitted for possible open access publication under the terms and conditions of the Creative Commons Attribution (CC BY) license (http://creativecommons.org/licenses/by/4.0/).

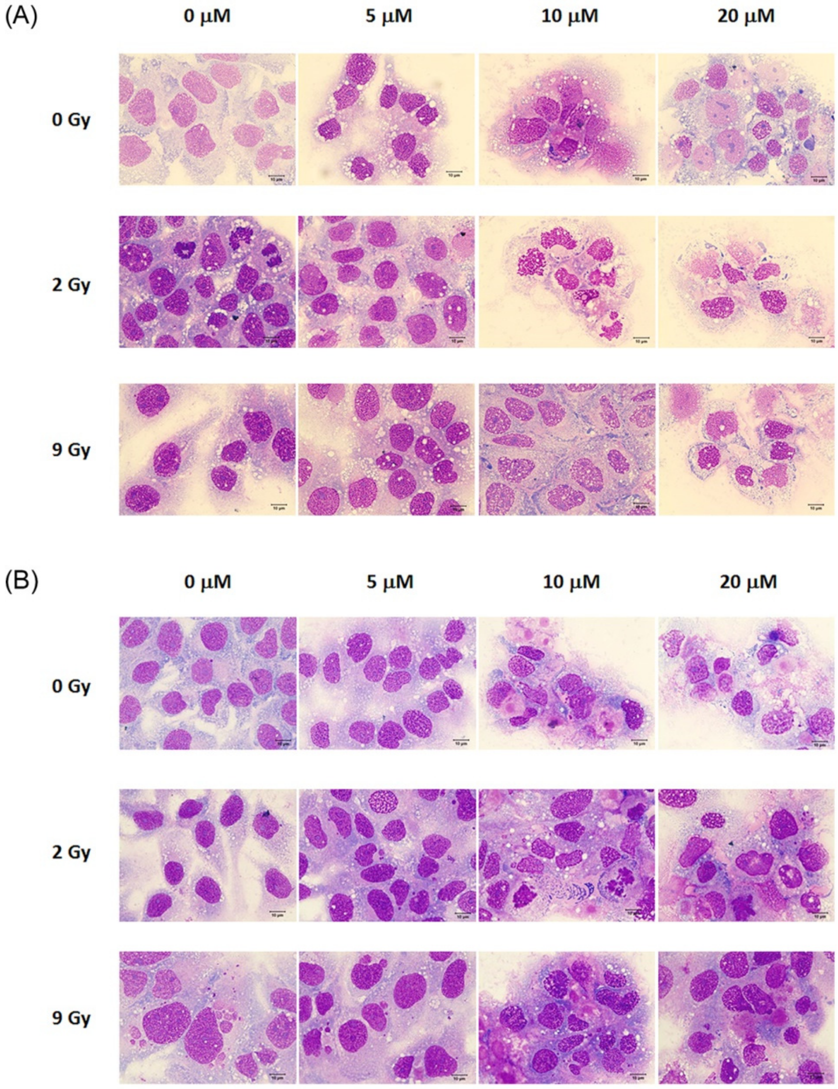

Figure S2. Huh-7 cells were treated with regorafenib at concentrations of 0, 5, 10, or 20 μmol/L (μM) either (A) 1 hr after irradiation (concurrent group) or (B) 24 hr after irradiation (sequential group) with sham RT (RT0 Gy), 2 Gy (RT2 Gy) and 9 Gy (RT9 Gy). Cell shrinkage and pyknosis varied in a dose-dependent manner with regorafenib and RT. Significant numbers of apoptotic bodies were observed in the sequential regimen of RT9 Gy. Cytoplasmic vacuoles were more clearly observed in the concurrent regimen. Additionally, cell swelling, the formation of cytoplasmic vacuoles and cytoplasmic blebs, and loss of cell membrane integrity were noted in the concurrent and sequential regimens of regorafenib (20 μM) with RT.

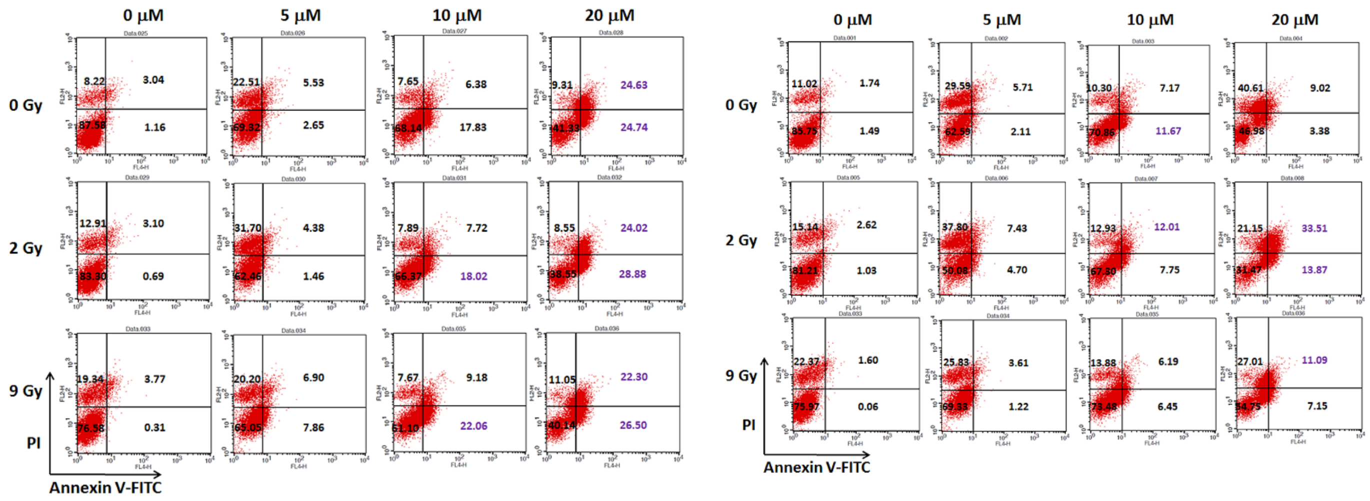

(**A**) (**B**)

Figure S3. Huh-7 cells were treated with regorafenib at concentrations of 0, 5, 10, or 20 μmol/L (μM). (A) Cells were stained with Annexin-V FITC and propidium iodide (PI). Annexin V(+)/PI(-) and Annexin V(+)/PI(+) were defined as early and late apoptotic cells, respectively. There were no obvious synergistic effects of apoptosis in the concurrent regimen. (B) RT followed by regorafenib enhanced the late apoptosis of Huh-7 cells.

Table S1. Interday and intraday assay precision (% RSD) and accuracy (% bias) values for the HPLC-UV determination of regorafenib in rat plasma.

**Nominal concentration (mg/mL)**	**Intraday**			**Interday**		
	**Observed concentration (mg/mL)**	**Precision (RSD,%)**	**Accuracy (Bias,%)**	**Observed concentration (mg/mL)**	**Precision (RSD,%)**	**Accuracy (Bias,%)**
0.1	0.125 ± 0.04	25.1	34.0	0.085 ± 0.01	−15.4	15.7
0.5	0.473 ± 0.07	−5.40	14.3	0.543 ± 0.04	8.65	7.23
1	1.056 ± 0.05	5.62	5.00	1.013 ± 0.07	1.35	7.12
5	4.928 ± 0.20	−1.45	3.96	4.995 ± 0.05	−0.10	1.01
10	9.989 ± 0.20	−0.11	1.98	9.933 ± 0.11	−0.67	1.15
25	25.04 ± 0.08	0.16	0.32	25.034 ± 0.04	0.13	0.16

Data are expressed as the mean ± S.D. (n = 6). Precision (% RSD) = S.D./Cobs*100. Accuracy (% bias) = (Cobs-Cnom)/Cnom × 100.
